# Clinicopathological significance of HSP70 expression in gastric cancer: a systematic review and meta-analysis

**DOI:** 10.1186/s12876-021-01990-4

**Published:** 2021-11-22

**Authors:** Xiaolu Wang, Li Xie, Lijing Zhu

**Affiliations:** The Comprehensive Cancer Centre of Drum Tower Hospital, Medical School of Nanjing University & Clinical Cancer Institute of Nanjing University, Nanjing, China

**Keywords:** Heat shock protein 70, Biomarker, Gastric cancer, Clinicopathological significance, Meta-analysis

## Abstract

**Background:**

Heat shock protein 70 (HSP70) has been associated with the clinicopathological characteristics and prognosis of many cancers types, implying that it is a potential cancer biomarker. However, no consensus has been reached regarding its clinicopathological and prognostic significance in patients with gastric cancer. To address this gap, we performed a systematic review and meta-analysis.

**Methods:**

We searched PubMed, Embase, and the Cochrane Library for full-text literature according to the eligibility criteria. We used the odds ratio and hazard ratio as the suitable parameters to evaluate the clinicopathological and prognostic significance of HSP70. The statistical analysis was performed using STATA 15.0.

**Results:**

After inclusion and exclusion of studies based on the eligibility criteria, data of 1,307 patients with gastric cancer from 9 studies were finally included. The pooled outcomes implied that HSP70 expression was significantly correlated with higher differentiation degrees, intestinal gastric cancer, and lymphovascular invasion but not with age, gender, depth of invasion, *Helicobacter pylori* infection, lymph node invasion, TNM stages, and metastasis. The pooled HR showed no significant correlation between HSP70 expression and overall survival of gastric cancer patients.

**Conclusions:**

Our meta-analysis showed that HSP70 plays a complicated role in the development of gastric cancer. It may be directly engaged in tumour differentiation and distant invasion but cannot be considered a biomarker for predicting the prognosis of gastric cancer.

## Background

Gastric cancer is one of the most life-threatening cancers, with high incidence and mortality rates worldwide [1]. The estimated number of case reported annually in China is 680,000, and the incidence rate continues to increase [2]. Despite advances in diagnostic and treatment strategies, the prognosis of patients with advanced gastric cancer remains poor [3]. The 5-year survival rate in patients with stage IV gastric cancer is approximately 4% [4]. Numerous studies have been conducted to understand the tumour microenvironment of gastric cancers. New biomarker-based treatment methods have shown promising results for improving the prognosis. Therefore, identifying strong biomarkers is important for accurately predicting the clinicopathological features and prognosis of gastric cancer.

Heat shock proteins (HSPs) are indispensable for haemostasis and protection and are produced by cells under stressful conditions. HSPs have been identified as biomarkers in almost all types of cancers including gastric cancer and are known to participate in tumour cell progression, aggressiveness, and treatment resistance [5]. They are classified into different families according to their molecular weight. HSP70 belongs to the most abundant family of HSPs and are primarily involved in the folding of denatured proteins. It also inhibits caspase-dependent and -independent apoptotic pathways [6, 7]. Increasing evidence has shown that HSP70 is related to the progression and development of various cancers, including cervical carcinoma, bladder urothelial carcinoma, colorectal cancer, and breast cancer [8–11]. Its prognostic effect has been confirmed in some solid tumours such as bladder, breast, and cervical cancers [12, 13]. Several studies have investigated the relationship between gastric cancer and HSP70 expression; however, the clinicopathological and prognostic significance of HSP70 remain unclear.

Hence, we performed a systematic review and meta-analysis to help resolve this issue. We investigated the correlation between HSP70 expression and the main clinicopathological features of gastric cancer. We also evaluated the prognostic value of HSP70 in patients with gastric cancer.

## Methods

This meta-analysis was performed according to the Preferred Reporting Items for Systematic Reviews and Meta-Analysis [14] and the Meta-analysis of Observational Studies in Epidemiology group guidelines [15].

### Search strategy

PubMed, Embase, and the Cochrane Library were searched for eligible studies by using the following keywords: ʻheat shock protein70 OR HSP70ʼ (all fields) AND ʻgastric OR stomachʼ (all fields) AND ʻtumor OR tumour OR neoplasm OR cancer OR carcinomaʼ (all fields). The search was conducted until 10 May, 2021, with no lower date limit. Additionally, the titles, abstracts, and full texts were manually searched using the references of the obtained literature to identify potentially relevant studies.

### Inclusion and exclusion criteria

The eligibility of studies was determined using the following inclusion criteria: 1) studies pertaining to gastric cancer and HSP70; 2) studies involving the detection of positive expression of HSP70 without using other biomarkers; 3) inclusion of sufficient data for estimating the prognostic effect of HSP70 and its association with major clinicopathological characteristics of gastric cancer in the original studies; and 4) inclusion of only the latest or most comprehensive study despite the availability of two or more studies investigated the same cohort of patients. The exclusion criteria were as follows: 1) animal trials, case reports, reviews, and conference abstracts; 2) articles providing insufficient data for HSP70 expression in gastric cancer.

### Data collection and quality assessment

A Microsoft Office Excel spreadsheet was created to tabulate the following information: author’s first name, year, nationality, number of samples, number of patients with positive expression and negative expression of HSP70, detection method, cut-off value, histological information, positive site, outcome statistics with the corresponding 95% confidence interval (CI), and follow-up information. When an article included both univariate and multivariate analyses, only the latter was used. The quality of each original study was assessed using the Newcastle–Ottawa Scale (NOS) [16], which is based on a ‘star system’ rated on a scale from 0 (lowest rating) to 9 (highest rating). Eligible studies with more than 6 stars were considered high-quality studies.

### Statistical analysis

ORs with 95% CI were used to evaluate the relationship between HSP70 expression and clinicopathological characteristics in patients with gastric cancer. To determine the association between HSP70 expression and the prognosis of patients with gastric cancers, HRs with 95% CIs were used as the summarised estimates. If such data were not directly found in the study and could only be taken from the figures, they were extracted using Engauge Digitizer 4.1 (http://sourceforge.net) from the Kaplan–Meier survival curves according to the guidelines established by Tierney et al. [17]. The level of heterogeneity among the studies was tested using the Q and I^2^ tests. A random-effect model was used to evaluate the data if the *P* value was < 0.10 or the I^2^ value was > 50%. Otherwise, a fixed-effect model was used [18]. Sensitivity analysis was also performed to determine the stability of aggregated results. In case of no substantial variations, our meta-analysis results could be considered stable [19]. The potential for publication bias was detected using the Begg’s test and Egger’s test. A *P* value of < 0.05 was considered to indicate a significant bias. All the analyses were performed using Stata 15.0 (StataCorp LP, College Station, TX, USA).

## Results

### Study selection and characteristics

**Fig. 1:**
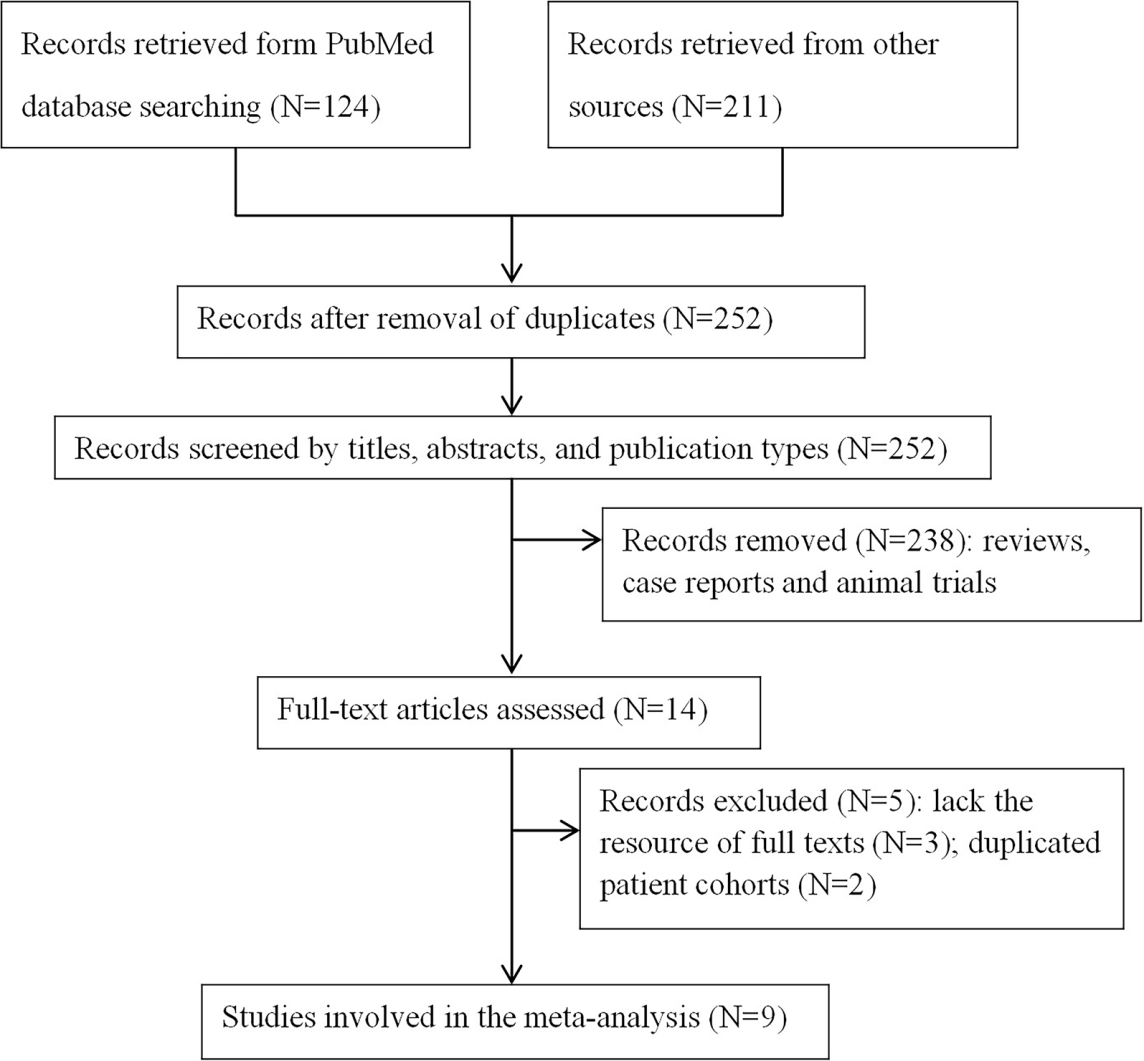
Flow chart outlining the selection of studies. Ultimately, 9 studies were selected to explore the clinicopathological and prognostic significance in patients with gastric cancer

Of a total of 335 studies initially retrieved from the 3 databases, 252 studies were selected after removing duplicates. The potential studies were screened based on the title, abstract, and publication type, and 238 studies were removed based on the exclusion criteria. Of the remaining 14 studies, 3 studies lacked full texts and 2 studies had investigated the same cohort patients as other studies. Finally, 9 studies were included for the meta-analysis [20–28] (Fig. 1). In total, 1307 patients from Jordan, China, Japan, Korea, Germany, and Turkey were included. Nine studies had reported the clinicopathological features of HSP70 [20–28], and 7 studies had investigated the prognostic role of HSP70 [20–25, 27]. Other features of the involved studies are summarised in Table 1. To determine HSP70 expression, 8 studies had used the immunohistochemical analysis [20–23, 25–28], whereas one study [24] had used flow cytometry. The average NOS score was 7.2 (range, 6–8), indicating that the quality of all the studies was good.

**Table 1:** Characteristics of included studies

Study	Country	Sample number (PE/NE)	Histology (intestinal/diffuse/mixed)	Detecting method	Cut-off value	Positive site	Investigating fields		Estimates	Follow-up (maximum)	NOS score
							CP features	Prognosis			
Bodoor et al. [20]	Jordan	87(18/69)	49/38/–	IHC	20% staining	Cytoplasm,Nucleus	√	√	OR,HR	88 months	7
Chid et al. [28]	China	50(39/11)	–	IHC	5% staining	Cytoplasm,Nucleus	√		OR	–	6
Ando et al. [21]	Japan	182(137/45)	–	IHC	70% staining	Cytoplasm	√	√	OR,HR	NR	7
Lee et al. [22]	Korea	172(50/122)	89/83/–	IHC	10% staining	Cytoplasm,Nucleus	√	√	OR,HR	NR	8
Kang et al. [23]	Korea	458(155/303)	239/219/–	IHC	50% staining	Cytoplasm	√	√	OR,HR	83.8 months	8
Pfister et al. [24]	Germany	19(7/12)	–	Flow Cytometry	10% staining	NR	√	√	OR,HR	50.4 months	6
Isomoto et al. [25]	Japan	81(55/26)	–	IHC	10% staining	Cytoplasm,Nucleus	√	√	OR,HR	NR	8
Canöz et al. [26]	Turkey	94(87/7)	56/21/17	IHC	5% staining	Cytoplasm	√		OR	–	7
Maehara et al. [27]	Japan	164(82/82)	–	IHC	19% staining	Cytoplasm,Nucleus	√	√	OR,HR	NR	8

*PE* positive expression; *NE* negative expression; *CP* clinicopathological; *IHC* immunohistochemistry; *OR* odds ratio; *HR* hazard ratio; *NR* not reported

### Association between HSP70 expression and the clinicopathological characteristics of gastric cancer

**Fig. 2:**
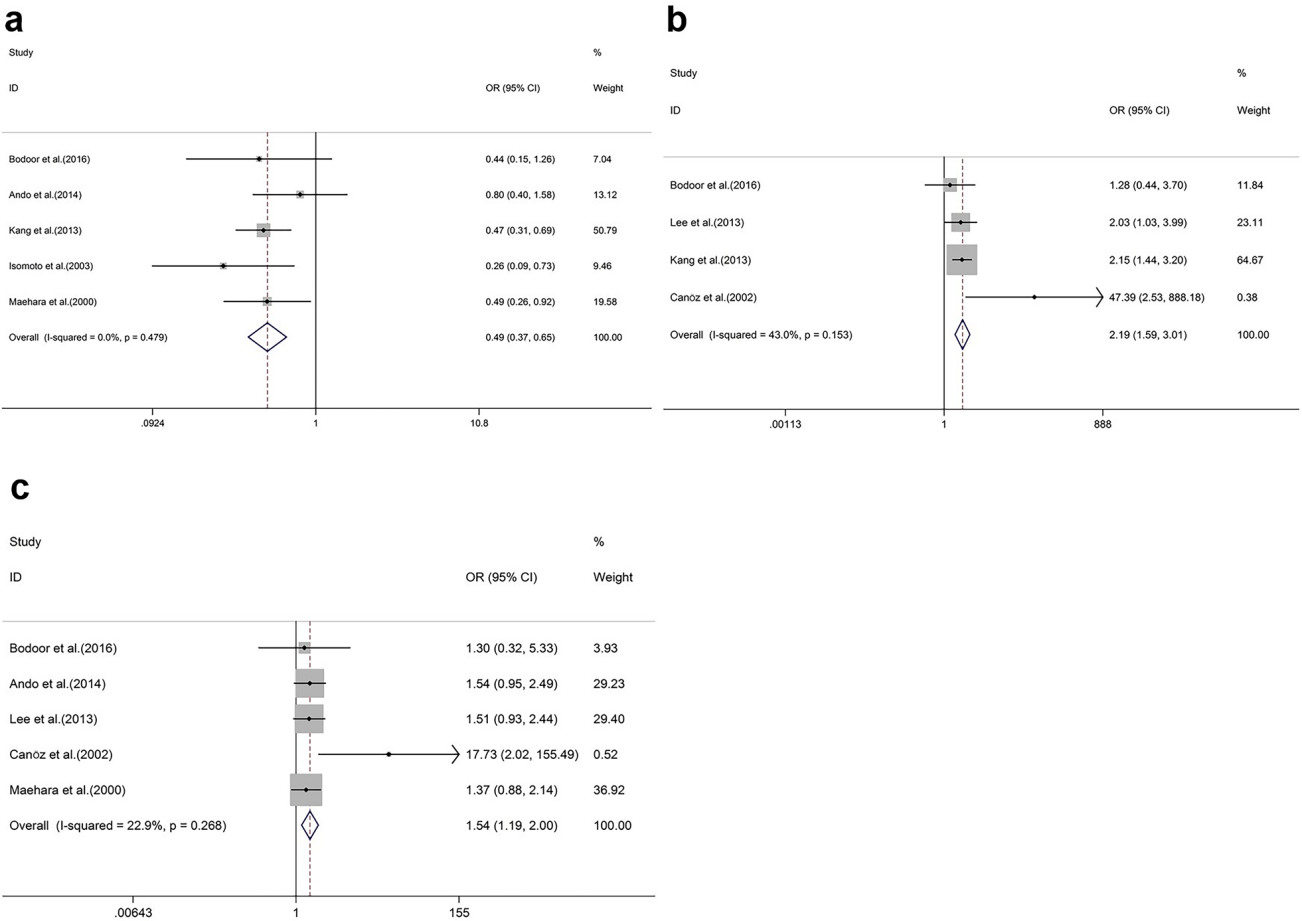
Pooled analyses for assessing the associations between HSP70 expression and **a** differentiation degrees, **b** histological subtypes and **c** lymphovascular invasion in patients with gastric cancer. *HSP70* heat shock protein 70

**Fig. 3:**
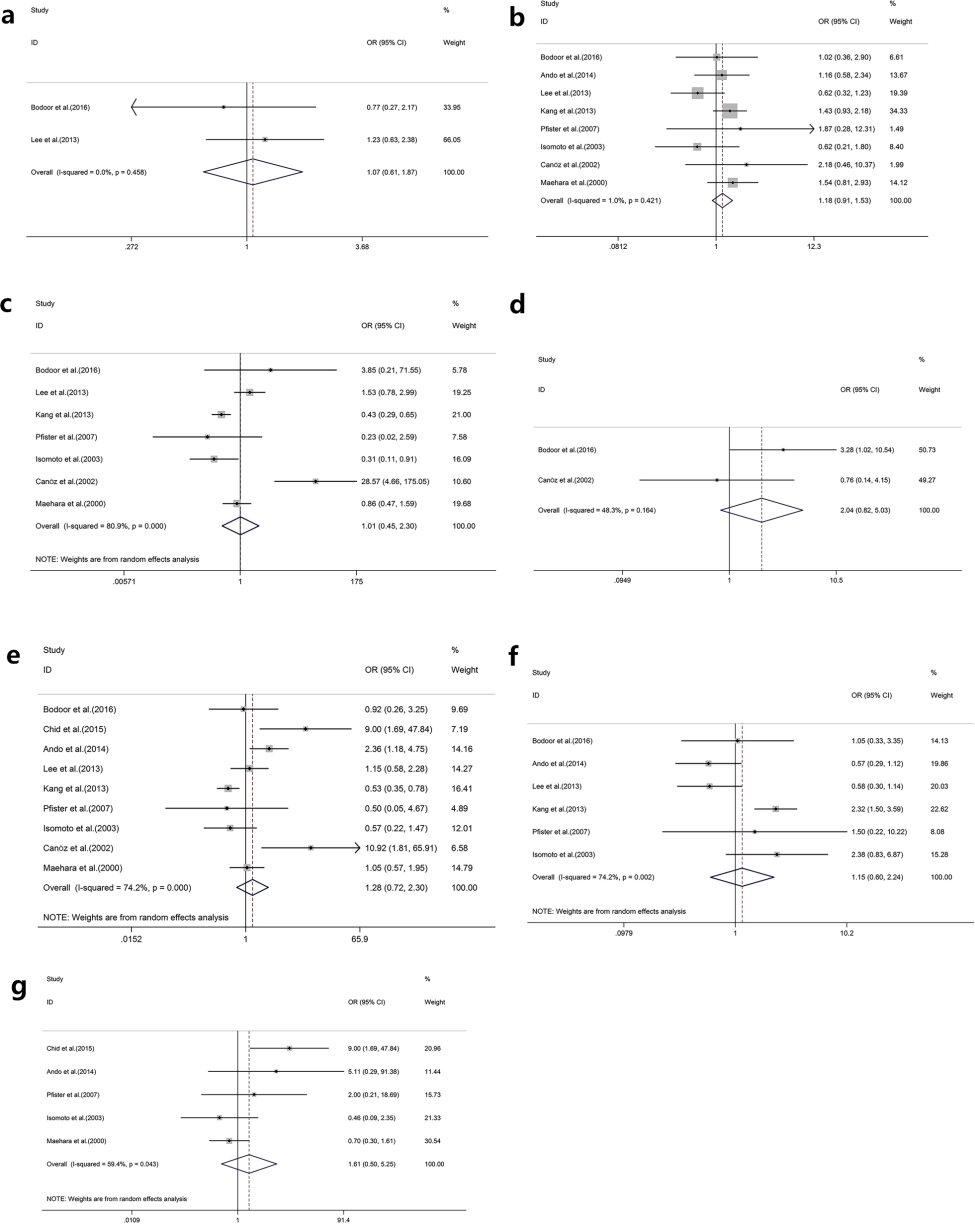
Pooled analyses for assessing the associations between HSP27 expression and **a** age, **b** gender, **c** depth of invasion, **d** H.pylori infection, **e** lymph node invasion, **f** TNM stages and **g** metastasis. *HSP70* heat shock protein 70

Demographic data were collected from each finalised article to calculate the corresponding OR, with 95% CI showing the HSP70 expression frequency based on different clinicopathological features. These parameters included ages, genders, depth of invasion, differentiation degrees, *Helicobacter pylori* infection, histological subtypes, lymph node invasion, TNM stages, lymphovascular invasion, and metastasis. Stage I–II tumours were defined as low TNM stages, whereas stage III–IV tumours were defined as high TNM stages. By comparing these ORs with 95% CI of each clinicopathological feature, we discovered that the positive expression of HSP70 was significantly correlated with a higher differentiation degree (OR = 0.49; 95% CI = 0.37–0.65; *P* < 0.001; I^2^ = 0.0%, *P* = 0. 479; Fig. 2a; Table 2), intestinal gastric cancer (OR = 2.19; 95% CI = 1.59–3.01; *P* < 0.001; I^2^ = 43.0%, *P* = 0.153; Fig. 2b; Table 2), and lymphovascular invasion (OR = 1.54; 95% CI = 1.19–2.00; *P* = 0.001; I^2^ = 22.9%, *P* = 0.268; Fig. 2c; Table 2). However, the results showed a nonsignificant correlation between HSP70 expression and clinical features such as age (OR = 1.07; 95% CI = 0.61–1.87; *P* = 0.809; I^2^ = 0.0%, *P* = 0.458; Fig. 3a; Table 2), sex (OR = 1.18; 95% CI = 0.91–1.53; *P* = 0.217; I^2^ = 1.0%, *P* = 0.421; Fig. 3b; Table 2), depth of invasion (OR = 1.01; 95% CI = 0.45–2.30; *P* = 0.975; I^2^ = 80.9%, *P* < 0.001; Fig. 3c; Table 2), *H. pylori* infection (OR = 2.04; 95% CI = 0.82–5.03; *P* = 0.123; I^2^ = 48.3%, *P* = 0.164; Fig. 3d; Table 2), lymph node invasion (OR = 1.28; 95% CI = 0.72–2.30; *P* = 0.399; I^2^ = 74.2%, *P* < 0.001; Fig. 3e; Table 2), TNM stages (OR = 1.15; 95% CI = 0.60–2.24; *P* = 0.672; I^2^ = 74.2%, *P* = 0.002; Fig. 3f; Table 2), and metastasis (OR = 1.61; 95% CI = 0.50–5.25; *P* = 0.426; I^2^ = 59.4%, *P* = 0.043; Fig. 3g; Table 2).

**Table 2:** Meta-analysis of the clinicopathological significance of HSP70 expression in patients with gastric cancer

Clinicopathological features	Reference count	Heterogeneity (I-squared, *p*)	Model	OR with 95% CI	*P* value	Conclusion
Age (≤ 60 years vs > 60)	2	0.0%, 0.458	Fixed	1.07(0.61–1.87)	0.809	Not significant
Gender (male vs female)	8	1.0%,0.421	Fixed	1.18(0.91–1.53)	0.217	Not significant
Depth of invasion (T3/T4 vs T1/T2)	7	80.9%, < 0.001	Random	1.01(0.45–2.30)	0.975	Not significant
Differentiation degrees (low vs high)	5	0.0%, 0. 479	Fixed	0.49(0.37–0.65)	< 0.001	Significant
H.pylori infection (positive vs negative)	2	48.3%, 0.164	Fixed	2.04(0.82–5.03)	0.123	Not significant
Histological subtypes (intestinal vs diffuse)	4	43.0%, 0.153	Fixed	2.19(1.59–3.01)	< 0.001	Significant
Lymph node invasion (positive vs negative)	9	74.2%, < 0.001	Random	1.28(0.72–2.30)	0.399	Not significant
TNM stages (I/II vs III/IV)	6	74.2%, 0.002	Random	1.15(0.60–2.24)	0.672	Not significant
Lymphovascular invasion (positive vs negative)	5	22.9%,0.268	Fixed	1.54(1.19–2.00)	0.001	Significant
Metastasis (positive vs negative)	5	59.4%,0.043	Random	1.61(0.50–5.25)	0.426	Not significant

*HSP70* heat shock protein 70; *OR* odds ratio; *CI* confidence interval

### The prognostic value of HSP70 expression in patients with gastric cancer

**Fig. 4:**
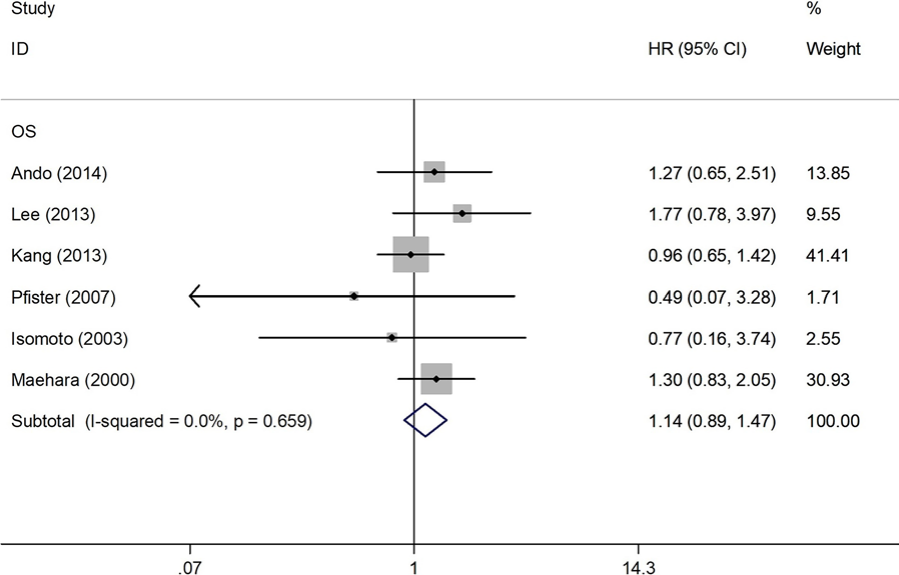
Pooled analyses for assessing the prognostic value of HSP70 expression for OS in patients with gastric cancer. HSP70 heat shock protein 70, OS overall survival

The association between HSP70 expression and patient survival had been explored in 7 studies, of which 6 studies had calculated OS [21–25, 27] and one had reported disease-free survival(DFS) [20]. The pooled HR of the former 6 articles was 1.14 (95% CI 0.89–1.47; *P* = 0.3; I^2^ = 0.0%, *P* = 0.659; Fig. 4), indicating no significant association between HSP70 expression and overall survival (OS) of patients with gastric cancer. However, further evaluation of the correlation between HSP70 expression and DFS was not pursued because of the lack of adequate number of studies and the availability of results.

### Sensitivity analysis

We performed the sensitivity analyses to determine both the clinicopathological and prognostic significance of HSP70 expression in gastric cancer (the derived forest plots are not shown). Eventually, a slightly high heterogeneity was found only between two studies[21, 28] evaluating the association between HSP70 expression and metastasis (I^2^ = 59.4%, *P* = 0.043). Therefore, the conclusion that the HSP70 expression is not related to metastasis may be unreliable. The strong stability of our meta-analyses for other clinicopathological features and the prognostic value of HSP70 in patients with gastric cancer were confirmed through further sensitivity analyses.

### Publication bias

**Fig. 5:**
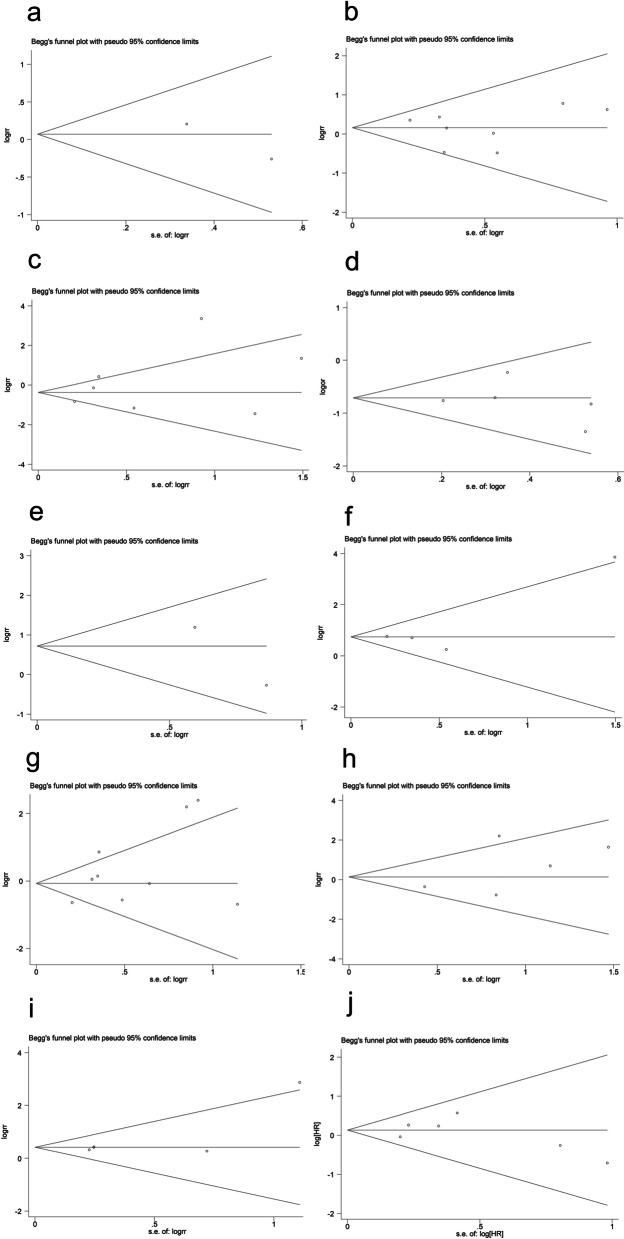
Funnel plot of studies assessing publication bias of the relationship between HSP70 expression and **a** age, **b** gender, **c** depth of invasion, **d** differentiation degrees, **e** H.pylori infection, **f** histological subtypes, **g** lymph node invasion, **h** metastasis, **i** lymphovascular invasion, **j** prognostic significance

No evidence for potential publication bias was found in this meta-analysis. The funnel plots obtained from the Begg’s test are shown in Fig. 5, and the corresponding Egger’s and Begg’s test *P* values are presented in Table 3. Although no remarkable publication bias was found in our meta-analysis, poor efficacies of both Begg’s and Egger’s tests were considered because of the scarcity of articles.

**Table 3:** Evaluations for the potential publication bias within the meta-analysis

Groups of outcomes	Reference count	Estimates	Begg’s test (*p* value)	Egger’s test (*p* value)	Publication bias
Age (≤ 60 years vs > 60)	2	OR with 95% CI	1.000	Not reported	Not significant
Gender (male vs female)	8	OR with 95% CI	0.536	0.786	Not significant
Depth of invasion (T3/T4 vs T1/T2)	7	OR with 95% CI	0.368	0.304	Not significant
Differentiation degrees (low vs high)	5	OR with 95% CI	1.000	0.745	Not significant
H.pylori infection (positive vs negative)	2	OR with 95% CI	1.000	Not reported	Not significant
Histological subtypes (intestinal vs diffuse)	4	OR with 95% CI	1.000	0.493	Not significant
Lymph node invasion (positive vs negative)	9	OR with 95% CI	0.466	0.101	Not significant
TNM stages (I/II vs III/IV)	6	OR with 95% CI	1.000	0.653	Not significant
Lymphovascular invasion (positive vs negative)	5	OR with 95% CI	0.221	0.180	Not significant
Metastasis (positive vs negative)	5	OR with 95% CI	0.221	0.295	Not significant
Prognostic significance	6	HR with 95% CI	1.000	0.781	Not significant

*OR* odds ratio; *HR* hazard ratio; *CI* confidence interval

## Discussion

Meta-analysis is a well-designed statistical method that combines data from similar articles to derive conclusive findings regarding an uncertain aspect. Predicting the prognosis of gastric cancer is difficult, and only a few biomarkers have been identified so far. To the best of our knowledge, this comprehensive meta-analysis is the first to investigate the relationship between HSP70 expression and the main clinicopathological characteristics of gastric cancer, as well as the role of HSP70 in the prognosis of gastric cancer. Our meta-analysis showed a significant association between HSP70 expression and several pathological features of gastric cancer such as higher differentiation degrees, intestinal gastric cancer, and lymphovascular invasion. However, the pooled results did not exhibit the prognostic value of HSP70 expression in terms of OS of patients with gastric cancer.

HSPs were first discovered in 1962 [29]. They act as molecular chaperones and assist in the folding of proteins under normal metabolic conditions and in amplifying the levels of repair and stabilisation of proteins in the face of molecular stress. In cancer, HSPs maintain homeostasis and promote cancer cell survival by inhibiting apoptotic induction [30, 31]. According to the molecular weight, HSPs have been classified into various families such as HSP110, HSP90, HSP70, HSP60, and small heat shock proteins [32]. HSP70 is a high-molecular-weight ubiquitous chaperone protein that regulates the cellular homeostasis by controlling protein folding, translocation, biogenesis, and degradation [33]. HSP70 expression is relatively lower in normal cells than in cancer cells [34]. Ando et al. hypothesised that tumour cells require higher levels of HSP70 than normal cells because of the carcinogenic stresses caused by the overexpression of abnormal oncoproteins and a high level of metabolism [35]. In experimental models, HSP70 overexpression increases the tumourigenicity of transformed cells, whereas HSP70 downregulation reduces the tumourigenicity of these cells [36]. Based on these findings, neutralising HSP70 has become a potential anticancer approach. The inhibitory molecule EGCG, a major flavonoid component of tea that inhibits the activities of HSP90 and HSP70, exhibits an antiproliferative effect on breast cancer cell lines and a xenograft model [37]. In another study, VER-155008 and PES reduced the viability of cancer cells by inhibiting HSP70 expression. VER-155008, an ATP-analogue, binds to the nucleotide-binding site of HSP70, whereas PES interacts with the substrate-binding domain of HSP70 [38]. The synthetic HSP70 inhibitor MAL3-101, a dihydropyrimidine, causes a reduction in the growth of xenotransplanted tumours in mice models, which suggests its role in the treatment of Merkel cell carcinoma [39]. In addition, HSP70 inhibitors have been reported to play a role in eliciting a greater response to neoadjuvant aromatase inhibitor treatment in breast cancer [40]. Despite the success of targeting HSP70 as a potential anticancer therapeutic approach, this strategy has some limitations. For example, because HSP70 is ubiquitously expressed in the physiological environment, its suppression can cause unexpected cytotoxicity in normal cells [36]. Thus, concerted efforts have been made to apply the targeted anticancer immunotherapy based on HSP70 expression.

HSP70 molecules interact with abnormal proteins such as mutated or altered oncogenes and tumour suppressor gene products [41], which are closely related to the progression of gastric cancer. HSP70 upregulation has been observed in tumour tissues and postulated to result from the stressful microenvironment of cancer cells; it might improve tumour cell survival by protecting proteins from degradation [42]. In our study, we reported that HSP70 expression was significantly correlated with the differentiation degree of gastric cancer. HSP70 expression in tumour cells increases in differentiated histiocytic lymphoma cells [43]. Therefore, we can assume that HSP70 is directly engaged in tumour differentiation. Differences in the HSP70 expression may be related to differences in pathogenesis. We also discovered that HSP70 expression was higher in the intestinal type than in the diffuse type of gastric cancer. The intestinal type is regarded to be the consequence of atrophic gastritis and intestinal metaplasia caused by environmental agents. Nevertheless, the diffuse type sometimes develops in the non-atrophic and non-metaplastic gastric epithelium with a strong genetic predisposition [44]. Thus, cancer cells of the intestinal type experience high levels of carcinogenic stress under environmental stimuli, which makes them more dependent on the high expression of HSP70. Finally, we found that HSP70 was correlated with lymphovascular invasion, indicating that HSP70 may be engaged in distant invasion. HSP70 is considered to play a substantial role in the recruitment and differentiation of inflammatory cells. HSP70 can also activate tumour immunity by stimulating innate immune mechanisms and strengthening the cross-presentation of tumour antigens to lymphocytes. Gastric cancer is closely associated with *H. pylori* infection, which can cause chronic inflammation [45]. Although no association between *H. pylori* infection and HSP70 expression was found in our study, an inflammatory microenvironment is believed to increase the HSP70 expression. Consequently, HSP70 was highly expressed in gastric cancer with lymphovascular invasion, playing an important part in tumour progression and metastasis. However, the underlying molecular mechanism remains unclear.

Based on the findings of these studies, some researchers have attempted to determine the potential prognostic significance of HSP70 expression in various types of cancers, including bladder, breast, and cervical cancer, melanoma, and acute myeloid leukaemia [12, 13, 46, 47]. HSP70 is associated with a poor prognosis in bladder, breast, and cervical cancers [12, 13]. The positive expression of HSP70 has also been found to be correlated with high metastatic potential and poor prognosis in malignant melanoma [46] and acute myeloid leukaemia [47]. However, a clinical study performed in patients with acute myeloid leukaemia demonstrated that patients with a high level of the anti-HSP70 antibody had longer survival [48]. Some studies have explored the potential prognostic significance of HSP70 expression in gastric cancer but reached a contradictory conclusion [20, 22, 24, 28, 35]. Incomplete references and differences in prognostic indicators may have considerably affected the accuracy of such studies. Therefore, in our meta-analysis, we used all the available evidence to perform a comprehensive assessment. However, our results did not reveal a significant relationship between HSP70 expression and the survival rate of patients with gastric cancer. The association of HSP70 with higher differentiation degrees, intestinal gastric cancer type, and lymphovascular invasion found in the present meta-analysis implies that HSP70 may play an important role in tumour differentiation and distant invasion. The mechanism of action of HSP70 in the development of gastric cancer is complex, which may partly explain the conclusion that HSP70 expression was not significantly associated with the survival rate of patients with gastric cancer.

In recent years, many studies have focused on exploring the role of HSP70 in GC treatment, and these studies have reported that HSP70 inhibition can enhance the sensitivity of GC cells to thermochemotherapy. The regulatory mechanism may be that Skp2 expression can influence the function of HSP70 [49]. HSP70 has also been reported to be related to apatinib resistance in patients with GC, indicating that apatinib resistance may be overcome by inhibiting HSP70 expression [50]. Furthermore, HSP70 has been considered as a potential therapeutic target and chemosensitiser in GC [51], and it has been developed into HSP vaccines for treating tumours such as melanoma, lymphoma, leukaemia, and glioblastoma [52]. The aforementioned results revealed the potential role of HSP70 in GC treatment. However, the specific mechanism of HSP70 in GC microenvironment remains unclear.

HSP70 may play multiple roles in the pathophysiology of gastric cancer. In cancer, HSPs maintain homeostasis and promote cancer cell survival by inhibiting apoptotic induction [31]. Some critical HSPs intricately regulate the fine balance between protective and destructive immunological responses within the tumour microenvironment [36]. The expression of HSP70 on the surface of tumour cells can confer an immunoregulatory role to this protein by increasing the ability of natural killer cells to lyse tumour cells, thereby improving the prognosis [53]. In addition, whether cancer cells expressing HSP70 on the surface are associated with a positive or negative prognosis may be determined by their metastatic pathways. Because of the limited number of current research samples, a consensus regarding the association between gastric cancer progression and HSP70 expression has not been reached, and it warrants further studies with a large sample size.

### Limitations of the study

The present study has several limitations that must also be considered. First, the sample size in the included studies was small. Second, differences in the ethnic backgrounds of patients with gastric cancer may have contributed to the inaccuracy of our findings. Third, for detecting HSP70 expression in the tumour tissues, different types of immunohistochemical antibodies and cut-off values were used in the included studies that might have contributed to some levels of heterogeneity. Fourth, the potential effect of different adjuvant treatments on gastric cancer patients may have also contributed to the inaccuracy of our results. Fifth, language bias may exist as we only included studies published in English. Sixth, we used Kaplan–Meier survival curves to obtain HRs and 95% CIs in case the study had not reported these data. In our future study, we aim to derive more convincing data by selecting more eligible studies.

## Conclusion

HSP70 expression is associated with higher differentiation degrees, intestinal gastric cancer type, and lymphovascular invasion. Additionally, HSP70 expression has no association with survival of patients with gastric cancer, which implies that HSP70 may play multiple roles in the pathophysiology of gastric cancer. To overcome the limitations of the present meta-analysis, more large-scale studies using standardised methods are required to be conducted in the future. In addition, studying the underlying mechanisms of HSP70 affecting tumour progression may contribute to the development of novel treatment strategies for gastric cancer. By applying evidence-based methods to more enrolled samples, this meta-analysis can provide insights into the clinicopathological role of HSP70 expression in gastric cancer.

## Data Availability

All data and materials during this study are presented within the manuscript.

## Abbreviations

HSP70: Heat shock protein 70
OR: Odds ratio
HR: Hazard ratio
HSPs: Heat shock proteins
NK: Natural killer
